# ADVANCE CARE PLANNING MOTIVATED BY THE COVID-19 PANDEMIC

**DOI:** 10.1093/geroni/igac059.2942

**Published:** 2022-12-20

**Authors:** Karuna Thomas, Fursan Sahawneh, Brian Carpenter

**Affiliations:** Washington University in St. Louis, St. Louis, Missouri, United States; Washington University in St. Louis, St. Louis, Missouri, United States; Washington University in St. Louis, Saint Louis, Missouri, United States

## Abstract

The COVID-19 pandemic has exposed older adults to complex healthcare situations, via personal experience or media stories about serious illness. Hearing about lengthy intubation, sedation, rapid decline, and distress at the end of life has the potential to prompt people to reevaluate their perspective on their own end-of-life care. This study explored advance care planning (ACP) among older adults and whether COVID-19 experiences altered their healthcare preferences and planning. One hundred and fifty-one respondents (M age = 71.2 yrs, range = 55–93) completed an online survey about ACP completion, ACP conversations, and life-prolonging interventions. Respondents were mainly female (78%), White (71%), and well educated (77% with at least a bachelor’s degree). A substantial proportion had not completed an advance directive (31%) or chosen a medical power of attorney (33%), and 78% of them intended to complete them in the next year or had initiated ACP. Among those who had completed ACP, a quarter intended to make changes, with 13% less open to life-prolonging medical treatments and 13% more open to them. Despite these intentions, a small proportion of people had ACP conversations since the start of the pandemic with their spouse/partner (37%), children (25%), siblings, (19%), friends (27%), primary care physician (16%), or other healthcare provider (12%). Results of this study suggest that now may be a critical moment to encourage older adults to have ACP conversations, in light of how their experience during the pandemic has motivated a reconsideration of treatment preferences.

